# Home-Monitoring Vision Tests to Detect Active Neovascular Age-Related Macular Degeneration

**DOI:** 10.1001/jamaophthalmol.2024.0918

**Published:** 2024-04-25

**Authors:** Ruth E. Hogg, Sobha Sivaprasad, Robin Wickens, Sean O’Connor, Eleanor Gidman, Elizabeth Ward, Charlene Treanor, Tunde Peto, Ben J. L. Burton, Paul Knox, Andrew J. Lotery, Michael Donnelly, Chris A. Rogers, Barnaby C. Reeves

**Affiliations:** 1 Center for Public Health, Queen’s University Belfast, Belfast, United Kingdom; 2 NIHR Moorfields Biomedical Research Center, Moorfields Eye Hospital NHS Foundation Trust, London, United Kingdom; 3 Bristol Trials Center, University of Bristol, Bristol, United Kingdom; 4 James Paget University Hospitals NHS Trust, Great Yarmouth, United Kingdom; 5 University of Liverpool, Liverpool, United Kingdom; 6 Department of Clinical and Experimental Sciences, Faculty of Medicine, University of Southampton, Southampton, United Kingdom

## Abstract

**Importance:**

Most neovascular age-related macular degeneration (nAMD) treatments involve long-term follow-up of disease activity. Home-monitoring would reduce the burden on patients and their caregivers and release clinic capacity.

**Objective:**

To evaluate 3 vision home-monitoring tests for patients to use to detect active nAMD compared with diagnosing active nAMD at hospital follow-up during the after-treatment monitoring phase.

**Design, Setting, and Participants:**

This was a diagnostic test accuracy study wherein the reference standard was detection of active nAMD by an ophthalmologist at hospital follow-up. The 3 home-monitoring tests evaluated included the following: (1) the KeepSight Journal (KSJ [International Macular and Retinal Foundation]), which contains paper-based near-vision tests presented as word puzzles, (2) the MyVisionTrack (mVT [Genentech]) vision-monitoring mobile app, viewed on an Apple mobile operating system–based device, and (3) the MultiBit (MBT [Visumetrics]) app, viewed on an Apple mobile operating system–based device. Participants were asked to test weekly; mVT and MBT scores were transmitted automatically, and KSJ scores were returned to the research office every 6 months. Raw scores between hospital follow-ups were summarized as averages. Patients were recruited from 6 UK hospital eye clinics and were 50 years and older with at least 1 eye first treated for active nAMD for at least 6 months or longer to a maximum of 42 months before approach. Participants were stratified by time since starting treatment. Study data were analyzed from May to September 2021.

**Exposures:**

The KSJ, mVT, and MBT were compared with the reference standard (in-hospital ophthalmologist examination).

**Main Outcomes and Measures:**

Estimated area under receiver operating characteristic curve (AUROC). The study had 90% power to detect a difference of 0.06, or 80% power to detect a difference of 0.05, if the AUROC for 2 tests was 0.75.

**Results:**

A total of 297 patients (mean [SD] age, 74.9 [6.6] years; 174 female [58.6%]) were included in the study. At least 1 hospital follow-up was available for 312 study eyes in 259 participants (1549 complete visits). Median (IQR) home-monitoring testing frequency was 3 (1-4) times per month. Estimated AUROC was less than 0.6 for all home-monitoring tests, and only the KSJ summary score was associated with lesion activity (odds ratio, 3.48; 95% CI, 1.09-11.13; *P* = .04).

**Conclusions and Relevance:**

Results suggest that no home-monitoring vision test evaluated provided satisfactory diagnostic accuracy to identify active nAMD diagnosed in hospital eye service follow-up clinics. Implementing any of these evaluated tests, with ophthalmologists only reviewing test positives, would mean most active lesions were missed, risking unnecessary sight loss.

## Key Points

**Question:**

Can any of the following visual function tests detect reactivation of neovascular age-related macular degeneration sufficiently well to enable monitoring at home to be implemented, including the KeepSight Journal (International Macular and Retinal Foundation), the MyVisionTrack (Genentech) vision-monitoring mobile app, and the MultiBit app (Visumetrics).

**Findings:**

In this diagnostic test accuracy study including 297 patients, none of the home-monitoring vision tests provided satisfactory diagnostic accuracy to identify active neovascular age-related macular degeneration diagnosed in follow-up clinics.

**Meaning:**

None of the tests evaluated are ready for implementation in a clinical situation.

## Teaser

This diagnostic test accuracy study investigates the ability of at-home vision-monitoring tests to detect reactivation of neovascular age-related macular degeneration.

## Introduction

Neovascular age-related macular degeneration (nAMD) is the leading cause of visual impairment in older adults.^1^ An active nAMD lesion causes exudation of fluid and/or blood at the macula, and patients experience distorted or blurred central vision. If untreated, the lesion causes a blind spot impairing the ability to do daily tasks requiring good central vision leading to reduced quality of life.^2^ Standard care is repeated intravitreal injections of inhibitors of vascular endothelial growth factor (anti-VEGF agents) to cause resolution of fluid and thereby improve or stabilize visual acuity.^3^ The usual care pathway (in the UK during study design phase) is initiated with a loading phase of 3 injections over 3 consecutive months; then, patients enter a maintenance phase, during which they are monitored at intervals in hospital eye clinics and usually retreated based on disease activity using either a treat-and-extend approach or pro re nata (prn or as needed). In either scenario, between-appointment intervals could be extended without concern about missing lesion reactivation or sight loss due to treatment delay.

A monitoring visit in a hospital eye clinic typically involves a health care professional deciding a treatment plan based on whether active nAMD is identified from changes in visual acuity, optical coherence tomography (OCT) scans of the macula, and ocular examination. Active lesions require treatment, whereas inactive ones do not. Monitoring intervals are determined by the treatment strategy of the clinician. Most patients will require many years of monitoring in hospital eye clinics even if they experience longer treatment-free periods because the risk of asymptomatic relapse is high.^4^ These monitoring appointments cause a significant burden to both patients and hospital eye clinics.

Several home-monitoring vision tests have been developed that may detect active disease.^5,6,7,8^ If patients could self-monitor for reactivation of nAMD, clinic attendance might only be necessary when active disease was detected, providing a significant benefit to patients and the health system. The COVID-19 pandemic accelerated the testing and use of home-monitoring tests for patients with inactive nAMD.^9,10,11^ When designing our study, we found no robust diagnostic accuracy study of any home-monitoring test, analyzed and reported in line with the Standards for Reporting of Diagnostic Accuracy Studies (STARD) reporting guidelines, and virtually all of the studies included authors who had either involvement in the development of the test or a financial/patent interest.^12^ This study aimed to estimate the test accuracy of 3 tests to self-monitor reactivation of nAMD compared with the reference standard of detection of reactivation during hospital follow-up. The tests chosen spanned a range of complexity and cost: 1 used paper and pencil, and 2 used modern information technology, implemented as software apps on an iPod Touch (Apple), hereafter referred to as a mobile operating system–based device.

## Methods

### Study Design

The Monitoring for Neovascular Age-Related Macular Degeneration Reactivation at Home (MONARCH) study was a multicenter, diagnostic accuracy cohort study to estimate the performance of 3 home-monitoring vision tests in detecting nAMD activation in patients undergoing treatment for active nAMD. Additional details can be found in the published protocol^13^ and an article using qualitative methodology to capture patient acceptability during the study.^14^ Ethical approval was granted by the Northern Ireland Health and Social Care Research Ethics Committee A (Reference number: 17/NI/0235) on January 29, 2018, and our study design and execution adhered to the Declaration of Helsinki. This study followed the Standards for Reporting of Diagnostic Accuracy (STARD) reporting guidelines.

Data collection was planned before the index test and reference standard were performed for any participant. This was a pragmatic evaluation, embedded in usual clinical practice: there were no specific requirements regarding treatment regimens, and no additional training to assess disease activity was given to clinicians.

Study participants were recruited from 6 National Health Service eye clinics in the UK. Patients 50 years and older with at least 1 eye first treated for active nAMD for at least 6 months or longer to a maximum of 42 months before enrollment and currently being monitored were eligible (termed a study eye). A potential study eye in an eligible patient was excluded if (1) the vision was worse than Snellen 6/60, (2) the eye had ocular surgery in the previous 6 months, (3) the eye had a recorded refractive error stronger than −6 diopters, or (4) there was non-nAMD macular neovascularization detected. In addition, a participant was excluded if they were unable to do one or more of the proposed tests during the training session and were unable to understand English or had personal or home circumstances that were unsuitable for home testing. Recruitment was not selective but depended on researchers at sites being available to approach and invite patients. For consented patients, race and ethnicity were recorded as either multiracial/multiethnic, White, or not recorded. Participant ethnicity was self-identified.

### Patient Identification

Potential study participants were identified by local teams from established clinical databases of patients with nAMD and by reviewing outpatient clinic lists. Details including reason(s) for nonparticipation, data to monitor inequalities, and information about at least weekly current use of widespread technologies were collected. Willing and eligible participants provided written consent to participate in the study after a training session. To recruit a study population that evaluated home monitoring across the relevant time spectrum of monitoring, recruitment was stratified according to time since first treatment for nAMD in the study eye (time since the first-treated study eye for participants with 2 study eyes): (1) 6 to 17 months, (2) 18 to 29 months, and (3) 30 to 41 months. Participants who completed the study were allowed to keep the mobile operating system–based device and mobile broadband device as a thank you for their participation.

### Test Methods

#### Home-Monitoring Tests

During the application and commissioning stage of this study, the literature was searched, and all tests with longitudinal data (including usability data) in patients with nAMD were considered for inclusion. The ForeseeHome Device (Notal Vision)^15^ was also considered, but the owners declined an offer to take part. All devices were European Commission marked and used within their intended purposes. All the peer-reviewed literature available for the chosen tests included authors who had financial or other interests in the tests. Inclusion of the tests was dependent on the owners or developers having no input in the design, implementation, analysis, or reporting of the study. This condition formed part of contractual agreements between all parties in accordance with the guidance of the funder.

#### KeepSight Journal

**Figure 1.**
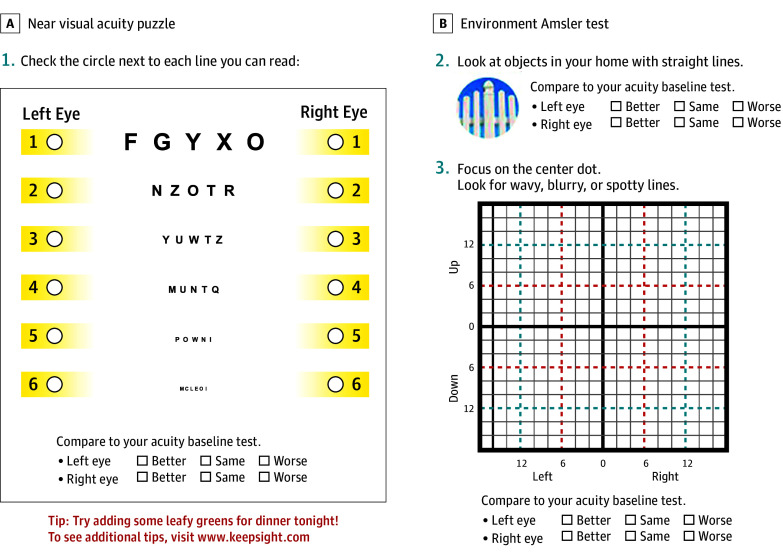
Example of a Page From the KeepSight Journal (KSJ [International Macular and Retinal Foundation]) A page in the KSJ contains a near visual acuity puzzle (A) and an environmental Amsler test including a modified Amsler grid (B).

The KeepSight Journal (KSJ [International Macular and Retinal Foundation]) is a paper-based booklet of near-vision tests for weekly monitoring, 1 eye at a time, using 3 monitoring strategies.^16^ It assesses near visual acuity using puzzles with diminishing font sizes (Figure 1A), self-reported distortion by encouraging patients to view objects with straight lines in the home (environmental Amsler)^17^ (Figure 1B), and distortion or scotomas using a modified Amsler chart or visual- and memory-stimulating grid (Figure 1C).^6^ Four raw test scores were generated for each testing occasion: near visual acuity worse than baseline, Amsler grid worse than baseline, household object appearance worse than baseline, ordinal near visual acuity (on a scale of 1-6, the number of font sizes displayed in each puzzle).

#### MyVisionTrack

**Figure 2.**
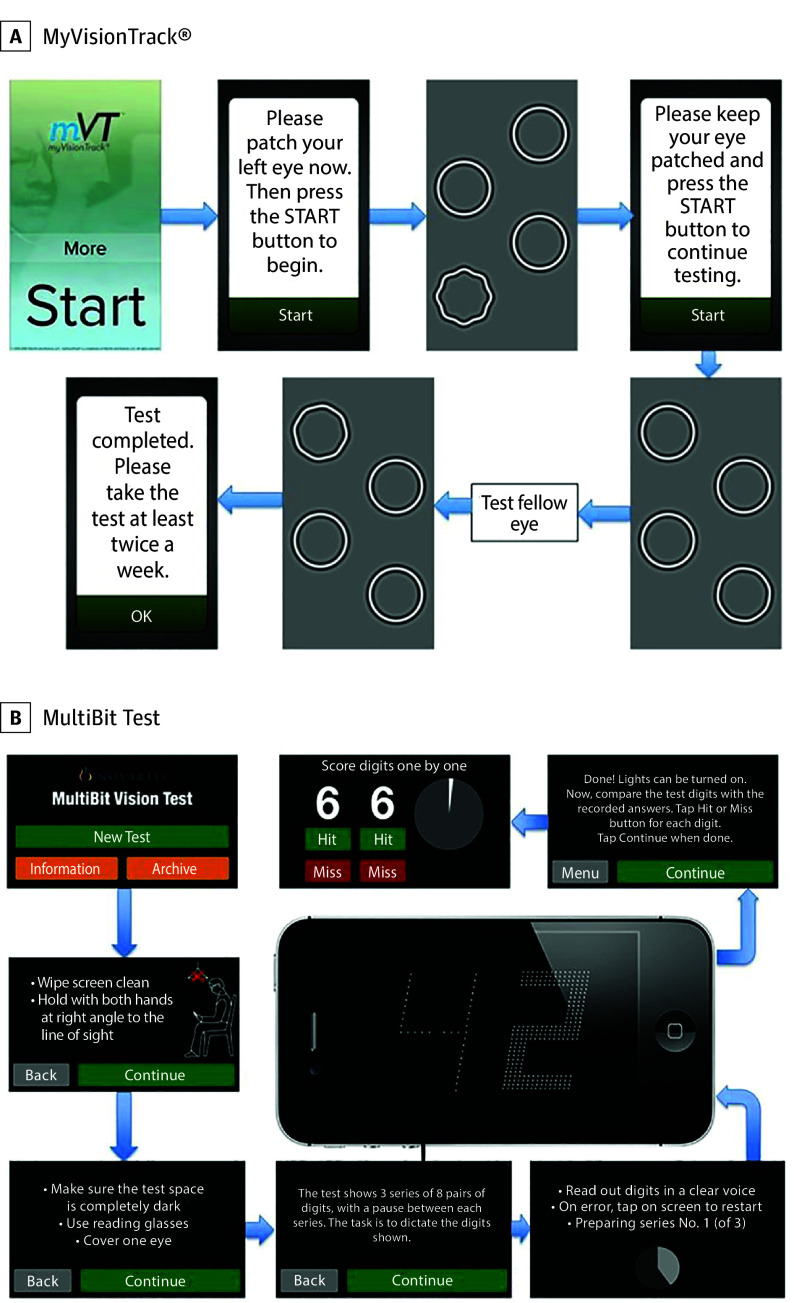
Example of the Content of Electronic Visual Games A, At-home vision monitoring mobile app, MyVisionTrack (mVT [Genentech]). B, Electronic software application, MultiBit Test (MBT [Visumetrics]).

The MyVisionTrack (mVT [Vital Art and Science]) vision-monitoring app, implemented on a mobile operating system–based device, presents a shape discrimination test that measures hyperacuity by displaying 4 circles, one of which is radially deformed (bumpy rather than perfectly circular).^18^ Viewing the display monocularly, the patient must identify the odd one out (Figure 2A). One raw test score was generated for each occasion when an eye was tested.

#### MultiBit

The MultiBit (MBT [Lars Frisén]) app, also on a mobile operating system–based device, presents a stimulus comprising receptive field–sized dots or rarebits (Figure 2B). Patients view pairs of numbers: they state the numbers that they see, and the numbers are then presented again at high contrast together with a recording of the patient’s responses. The patient scores their own performance. One raw test score was generated for each testing occasion.

### Training and Steps to Minimize Barriers

All participants who were willing in principle to participate were informed about the study and invited to attend a training session on how to use the 3 tests. Each participant was provided with a mobile operating system–based device, a lens cloth, an eye patch, stylus pen, a mobile broadband router for internet connectivity, and a mains micro–Universal Serial Bus charger to minimize barriers for self-monitoring at home (eFigure 1 in Supplement 1). The study management team provided a support telephone line during standard office hours. The team also made calls to participants if no app data had been received within 2 weeks of consent or 3 weeks after the previous test.^19^

### Reference Standard

The primary reference standard test was the reviewing ophthalmologist’s decision at a usual-care monitoring visit in the hospital eye clinic about the activity status of the nAMD lesion in the study eye. This decision was made based on usual-care tests, ie, clinical examination, visual acuity assessments, or OCT scans, without access to data from home-monitoring tests. The ophthalmologist recorded the decision as active, inactive, uncertain, or no lesion. The reference standard grouped uncertain with inactive judgments for analyses.

We also considered an alternative reference standard in secondary analyses, namely, a change in disease classification from inactive to active between pairs of monitoring visits. We specified this alternative reference because it represented how home-monitoring could be implemented, ie, discharge from hospital follow-up when a patient has inactive disease and recall only when home-monitoring test results diagnose reactivation.

### Follow-Up

The data from the mobile operating system–based device were transmitted automatically from the 2 apps to the study database via the mobile broadband device; completed KSJ booklets were designed for weekly testing and returned by mail every 6 months. Participants were asked to self-test weekly, and typically, several test results were available between attendances at usual-care monitoring visits. The primary analysis averaged all available test data between adjacent hospital monitoring visits.

MBT provided a score at the end of testing; however, no guidance was provided to either participants or study staff as to how to interpret this information. Further discussion on the impact of this feedback on participants can be found in our qualitative study results.^14^ Participants were followed up for at least 6 months. Reference data were collected at all usual-care hospital-monitoring clinics from the time of enrollment until a participant withdrew or data collection terminated.

#### Sample Size

Hospital-monitoring visits comprised the units of observation. We originally specified a sample size of at least 400 participants and a minimum follow-up of 12 months, generating about 2300 monitoring visits based on an average of 5 to 6 monitoring visits per participant. We assumed an effective sample size of 1200 monitoring visits because multiple monitoring visits by 1 participant did not represent independent observations, thereby diluting the nominal power to discriminate test performance. This effective sample size would have had 90% power to detect a difference of 0.06, or 80% power to detect a difference of .05, in the area under the receiver operating characteristic curve (AUROC) for 2 tests if the AUROC was greater than 0.75.^12^ However, we did not achieve that target, and with approval from the funder, we maximized power to address the original objectives by amending the protocol to reduce the minimum follow-up time to allow more patients to be recruited with fewer average visits per participant.

### Statistical Analysis

We excluded data from monitoring visits without an OCT (a common occurrence during the COVID-19 pandemic) because the absence of an OCT changed the reference standard or if there was no ophthalmologist’s decision on lesion activity status. Data on study eyes were included if there were at least 2 monitoring visits after enrollment with an ophthalmologist’s decision on lesion activity status and home-monitoring test data for at least 1 home-monitoring test during the interval between the preceding visit (ie, either the baseline visit or a preceding monitoring visit) and subsequent visit. As participants did not self-test with all 3 tests on every occasion, the number of study eyes varied across analyses. Uncertain (and 1 no-lesion) reference classifications were combined with inactive classifications for all analyses.

For the primary analysis, summary scores were derived by averaging (mean, median, or proportion of tests reported as worse) all raw scores accruing in the interval between visits. AUROC results were estimated by fitting predictors of the reference standard in logistic regression models. An AUROC was estimated first using only participants’ baseline data to predict the reference (the no-test model, fitting sex, stratum for time since first treatment for nAMD at baseline, age, visual acuity stratum at baseline, and log-transformed days since baseline monitoring visit). Separate models were then fitted for each test by adding the test summary score added (ie, estimating diagnostic accuracy adjusting for baseline data) and the incremental accuracy quantified; for the KSJ, all 4 summary scores were fitted at the same time. Interactions and combinations of summary test scores were not considered. Clustering of 2 study eyes within some participants, and visits within eyes, was considered through 3-level linear mixed modeling.

The primary analysis of test accuracy used the reference standard and all available home-monitoring data between pairs of complete monitoring visits. A sensitivity analysis (SA1) used the most recent data, ie, for only the 4 weeks preceding the second visit. A second sensitivity analysis (SA2) derived the reference standard from independent grading of OCTs by a Reading Center (NetwORC UK).^20^ A final analysis estimated AUROC results for the alternative reference standard of a change from inactive to active disease classification between monitoring visits.

Performance of the no-test and test models is described by AUROC estimates, odds ratios (ORs) for the test summary scores, and their respective 95% CIs. Sensitivity, specificity, and positive and negative predictive values of each test were reported with 95% CIs based on threshold scores that minimized misclassifications.^21^ A 2-sided *P* value <.05 was considered statistically significant. Study data were analyzed from May to September 2021; SAS software, version 9.4 (SAS Institute), was used for data handling/descriptive tables, and Stata, version 17 (StataCorp), was used for all analyses.

## Results

**Figure 3.**
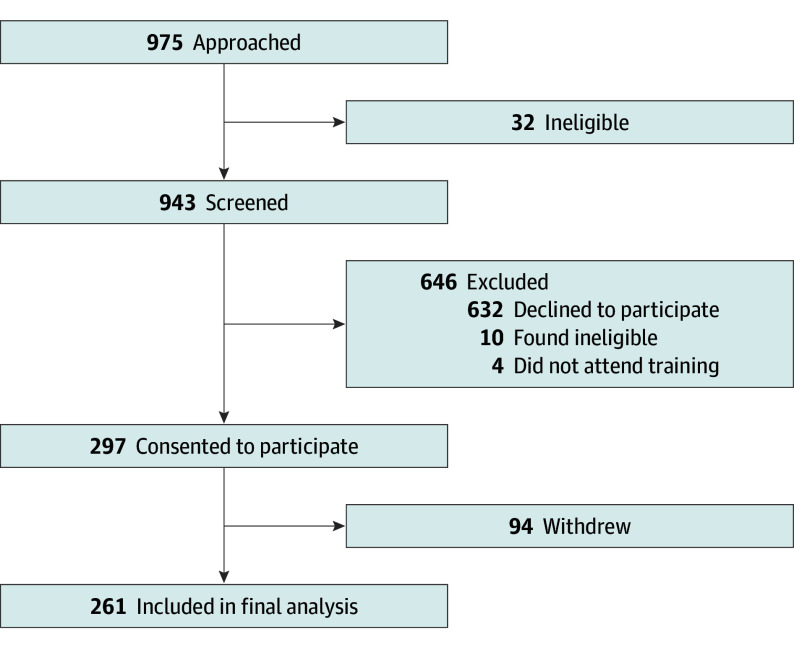
Chart Showing the Flow of Patients Through the Study

Participants were recruited from August 21, 2018, to March 31, 2020. Data were collected for all monitoring visits up to September 30, 2020. A total of 297 participants (mean [SD] age, 74.9 [6.6] years; 174 female [58.6%]; 123 male [41.4%]) provided written consent to participate. Consented patients identified with the following race and ethnicity categories: 3 multiracial/multiethnic (1%), 206 White (69%), and 88 (30%) had no ethnicity recorded. Some had no subsequent complete monitoring visits; hence, 261 patients contributed data to the analyses. The flow of patients in the study is shown in Figure 3. A total of 297 of 975 approached patients (30%) consented. Reasons for ineligibility at screening and training are shown in eTable 1 in Supplement 1. Overall, 94 of 297 patients (32%) withdrew (not due to the COVID-19 pandemic). Participants’ baseline characteristics are described in the Table. Most participants had substantial exposure to technology before entering the study. Median (IQR) home-monitoring testing frequency was 3 (1-4) times per month.

**Table.** Baseline Demographic Characteristics and Exposure to Technology of 297 Consented Participants

Baseline characteristic	No./total No. (%)
Sex	
Male	123/297 (41.4)
Female	174/297 (58.6)
Race and ethnicity	
Multiracial/multiethnic	3 (1)
White	206 (69)
Not recorded	88 (30)
Age, mean (SD), y	74.9 (6.6)
No. of participants with	
2 Study eyes^a^^,^^b^	60 (20.2)
1 Study eye (throughout)^c^	237 (79.8)
Visual acuity in study eye, mean (SD), ETDRS	72.9 (10.7)
Smoking history	
Current smoker	30/297 (10.1)
Ex-smoker (>1 mo)	137/297 (46.1)
Never smoked	130/297 (43.8)
Medical history	
Congestive cardiac failure	11/297 (3.7)
Myocardial infarction	19/297 (6.4)
Peripheral vascular disease	7/297 (2.4)
Cerebrovascular disease	21/297 (7.1)
Hypertension requiring treatment	158/297 (53.2)
Chronic pulmonary disease	28/297 (9.4)
Rheumatological disease	53/297 (17.8)
Renal disease	25/297 (8.4)
Liver disease	7/297 (2.4)
Neurological dysfunction	12/297 (4.0)
Malignancy	60/297 (20.2)
Diabetes, type 1	7/297 (2.4)
Diabetes, type 2	31/297 (10.4)
Other conditions that may affect ability to perform testing	15/297 (5.1)
Exposure to technology	
Television	294/296 (99.3)
Simple mobile phone	130/296 (43.9)
Smartphone	197/296 (66.6)
Tablet	196/296 (66.2)
Laptop/home computer	184/296 (62.2)
Internet at home	252/296 (85.1)
Email	213/296 (72.0)
Social media	97/296 (32.8)
TV streaming/on-demand services	146/296 (49.3)

Abbreviation: ETDRS, Early Treatment Diabetic Retinopathy Study.

^a^ Four of 50 participants with 2 study eyes at baseline had no follow-up hospital management visits.

^b^ Ten patients had eyes excluded at baseline (due to diagnosis of neovascular age-related macular degeneration within the last 6 months), which became eligible during follow-up and were included as study eyes thereafter; 3 of 10 patients had no follow-up hospital management visits.

^c^ A total of 34 participants with 1 study eye had no follow-up hospital management visits.

### Availability of Reference Standard

At least 1 complete monitoring visit after starting to use the home-monitoring tests and test data were available for 312 study eyes from 259 participants. Reference classifications for the study eye for all complete visits (n = 1549) were as follows: 932 active (60.1%), 576 inactive (37.2%), 40 uncertain (2.6%), and 1 no lesion (0.1%).

### Diagnostic Accuracy for the Primary Reference Standard

**Figure 4.**
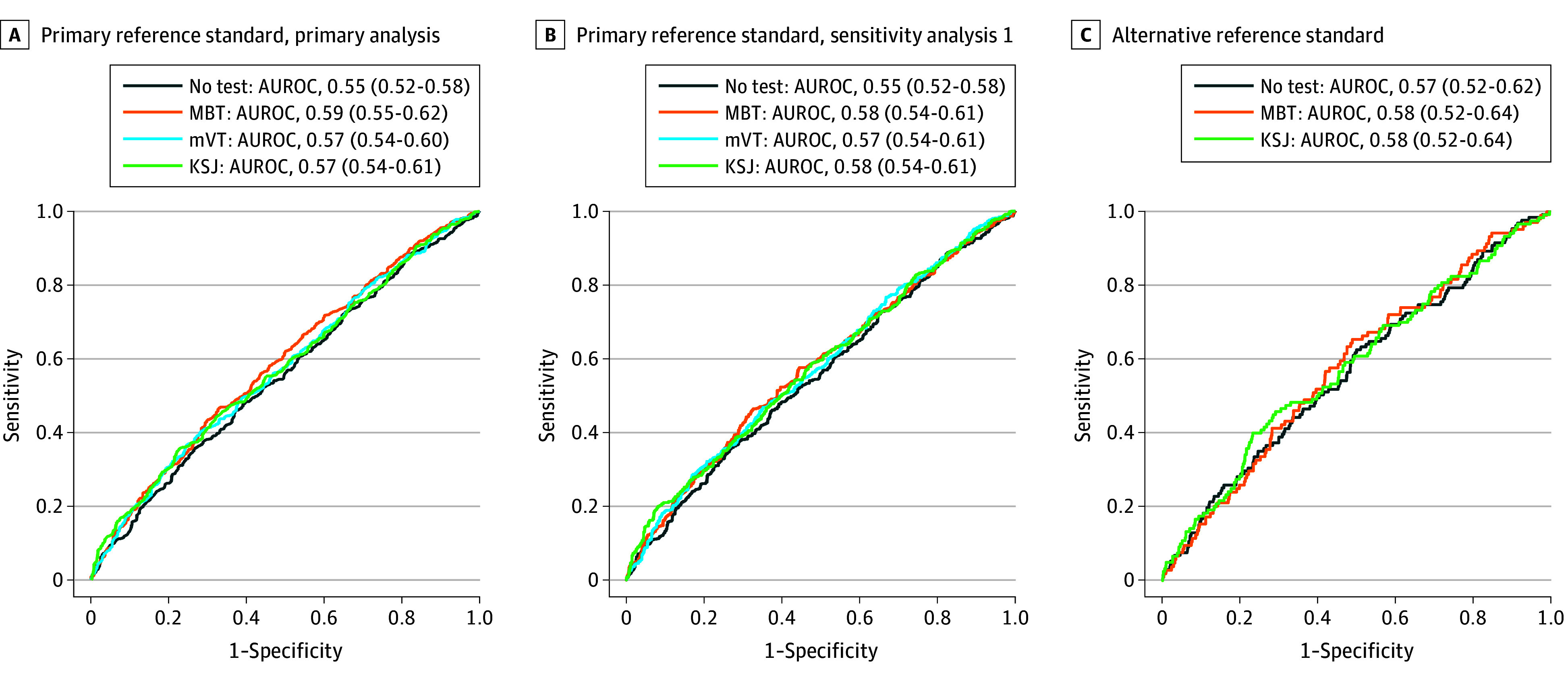
Diagnostic Accuracy Receiver Operating Characteristic Curves and Area Under the Receiver Operating Characteristic Curve (AUROC) A, Primary reference standard, using all test data available between visits (primary analysis). B, Primary reference standard, using test data from previous 4 weeks only (sensitivity analysis 1). C, Alternative reference standard (reading center–graded optical coherence tomography scans). The parentheses include the lower and upper range of the 95% CIs. KSJ indicates KeepSight Journal; MBT, MultiBit Test; mVT, MyVisionTrack.

Figure 4A shows ROC curves for the no-test model and models for each test. The curves were similar for the 4 models, with estimated AUROC results less than 0.6; sensitivities were approximately 0.4 and specificities approximately 0.7. Full model results are shown in eTable 2 in Supplement 1.

None of participant sex, age, time since first treatment at baseline, or visual acuity stratum at baseline significantly predicted lesion activity, irrespective of whether a home-monitoring summary score was included.

Among the home-monitoring summary scores, only the KSJ worse vision summary score showed a statistically significant association with lesion activity. Patients who reported their vision to be worse than baseline were more likely to have a lesion assessed as active compared with patients who reported the same or better vision (OR, 3.48; 95% CI, 1.09-11.13; *P* =.04; this OR magnitude represents a participant reporting worse vs same or better vision for all testing occasions between 1 visits). There were no adverse events from doing the home-monitoring tests. The reference was usual care.

### Sensitivity Analyses

When using test data for the preceding 4 weeks only (SA1), the baseline model results were the same as in the primary analysis because all predictors and the outcome were identical. ROC curves and AUROC results for summary test scores for all tests are shown in Figure 4B. As in the primary analysis, the AUROC values for the tests were only marginally larger than for the model that excluded the tests, with 95% CIs that overlapped across all models. Full model results can be examined in eTable 3 in Supplement 1. The results of the sensitivity analysis for the reference standard derived from independent grading of OCTs (SA2) are shown in eTable 4 and eFigure 2 in Supplement 1.

### Alternative Reference Standard (Transition From Inactive to Active Classification)

There were 1413 visit pairs for analysis, 131 (9.3%) of which described changes from inactive disease classification at the preceding visit to active disease classification at the subsequent visit (Figure 4C). Again, AUROC results for test models were only marginally larger compared with the no-test model, with 95% CIs that overlapped across all models. No significant associations were seen for any home monitoring test or other covariate. Full model results can be examined in eTable 5 in Supplement 1.

## Discussion

We believe that this was the first pragmatic, multicenter evaluation of the diagnostic accuracy of home-monitoring of visual function in patients with nAMD and reported according to STARD guidelines (eTable 6 in Supplement 1).^22^

### Diagnostic Accuracy of Home-Monitoring Tests

None of the tests had adequate diagnostic test accuracy for the primary outcome to enable patients to monitor their vision at home. This conclusion was unaltered by the sensitivity analyses. The alternative reference standard tried to address how home-monitoring might be implemented; diagnostic accuracy of the tests was similar, though imprecisely estimated due to the small number of pairs of visits documenting lesion reactivation.

### Findings in the Context of Existing Literature

One of the apps we studied (mVT) was introduced as a service quality improvement initiative at Moorfields Eye Hospital in London, one of the participating sites in our study. Although 417 patients were offered the app, only 166 (40%) became active and compliant users.^11^ The numbers of patients/eyes with active/inactive disease were not described, although sensitivity and specificity were reported to be 84.6% and 88.5%, respectively. In fact, these estimates appear to represent the positive predictive value (PPV; ie, 22 of 26 test positives had active disease) and 1 PPV (3 test positives had stable disease, ie, [26 − 3]/26); this estimate differs from the first because 1 patient had a retinal detachment. Applying plausible assumptions, eg, for the numbers of patients who had active disease and inactive disease when reviewed, leads to sensitivity and specificity estimates of approximately 19% and 92%, respectively.

Two other studies^9,10^ used the Alleye app (Oculocare). One study^10^ evaluated 245 patients undergoing treatment. Analysis focused on patients (85 eyes) who generated persistent alarms; of these, clinical review findings were available for 78, of which 28 were considered to have deteriorated (PPV = 36%). Again, applying plausible assumptions leads to sensitivity and specificity estimates of about 14% and 68%, respectively. The second study^9^ of this app included 73 eyes with nAMD or diabetic macular edema of 56 patients and investigated test scores against multiple clinical reviews (as in the MONARCH trial). The authors reported specificity of 94% and a PPV (based on 25 alarms) of 80%, from which we calculate a sensitivity of 14%.

All these studies were single center and did not report according to STARD guidelines,^22^ notably failing to provide a cross-tabulation of the index test results by the results of the reference standard and (correct) estimates of diagnostic accuracy and their precision (STARD items 23 and 24).

### Implications

Both app-based tests had formal spin-off companies, and at the time of trial setup, both had been acquired by pharmaceutical companies. Hence, despite having no diagnostic accuracy information, they were considered promising when the study was initiated. The findings of this study show that an independent, high-quality evaluation of diagnostic accuracy is essential before tests are implemented in clinical pathways. Most pilot studies describe average associations rather than the accuracy of prediction of the classification of individuals. Studies are typically designed to optimize test performance in populations or settings, resulting in biased estimates of test performance.

### Strengths and Limitations

This study has several strengths. Features to minimize bias were prioritized in the design and conduct of the study. Selection bias was avoided by using a cohort study design, and research teams were informed to invite consecutive eligible patients to participate and, hence, ensured a representative sample of patients. Assessment bias of the tests was avoided by ensuring that the tests were scored without knowledge of the results of the reference standard. Home-monitoring tests were performed before monitoring visits to avoid being influenced by the reference standard classification. The usual-care team in the monitoring clinics who assessed the reference standard was masked to data from home-monitoring tests.

Bias due to exclusion of participants or inappropriate intervals between the times of testing and the reference standard was avoided by ensuring that the analyses included all follow-up visits for which the reference standard was assessed. The first sensitivity analysis investigated the trade-off between the recency of test information and the precision of summary scores. We also accounted for all patients recruited into the study. We attempted to minimize inequality in access by providing the monitoring devices and an additional portable rechargeable/battery-powered wireless device (MiFi [Hutchison 3G]). All tests evaluated were developed independently of the study investigators, and the developers had no role in the conduct of the study or the analysis plan. The study has been reported in accordance with STARD guidelines (eTable 6 in Supplement 1).^22^

This study also has some limitations. The sample size was smaller than planned (less than half the target number of monitoring visits); nonetheless, 95% CIs for the AUROCs for the test models were narrow (± 0.04) and consistently overlapped the 95% CIs for no test, ruling out the possibly that any test provided adequate accuracy for diagnosing active nAMD to enable patients to be monitored at home. Test developers did not provide test thresholds; therefore, these could not be defined a priori; instead, we estimated the AUROCs. We did not compare AUROCs for tests due to their poor accuracy. We reported sensitivity and specificity based on the Youden index; acknowledging the latter takes no account of the relative clinical and other implications of false-positive and false-negative misclassifications. Sites were trained to provide information to participants about how to perform home monitoring, ie, training the trainers. The study had no control over monitoring visits, and participants are likely to have reported their subjective visual experience to their consultants, which may have influenced the reference standard. We also acknowledge potential variation in usual care between sites and how ophthalmologists assessed disease activity. Nearly one-third of participants withdrew over the duration of the study. Those continuing to test (and provide more data) may represent a selected subset. Withdrawals are dealt with more fully in another publication.^23^ We also recognize that we were comparing visual function tests against a retinal imaging reference standard which, although it most closely reflects the clinical reality, may not compare the same pathological components. Visual acuity assessed in the clinic may have been a more closely related comparison and is worth exploring in future studies.

## Conclusions

In this diagnostic test accuracy study, none of the following home-monitoring visual function tests detected reactivation of nAMD sufficiently well to enable monitoring at home to be implemented: KSJ, mVT, or MBT. Although none of the tests evaluated seem ready for implementation in a clinical situation, the findings do support rigorous evaluation of such technologies in the settings and populations for which they are intended before widespread implementation.

## Notes

Supplement 1. **eFigure 1.** Equipment Pack Given to Participants Included an iOS-Based Mobile Device, Eye Patch, Stylus, Screen Cloth, Laminated Instruction Card, Wireless Internet Device, 2 Charging Leads, and a Double USB Charger Plug **eFigure 2.** Receiver Operating Characteristic Curves and AUROCs (95% CIs) for Summary Test Scores for All Index Tests; Sensitivity Analysis 2 (Reference Standard Derived From Independent Grading of OCTs) **eTable 1.** Reasons for Being Unwilling in Principle to Participate **eTable 2.** Primary Analysis of the Diagnostic Accuracy of Baseline Information (“No Test”) and of the Summary Scores for Home-Monitoring Tests to Identify the Reference Standard of Lesion Activity in Study Eyes **eTable 3.** Model Results for Sensitivity Analysis 1 of the Diagnostic Accuracy of Baseline Information (“No Test”) and of the Summary Scores for Home-Monitoring Tests to Identify the Reference Standard of Lesion Activity in Study Eyes Used Home-Monitoring Data for Only the 4 Weeks Preceding the Hospital Monitoring Visit **eTable 4.** Model Results for Sensitivity Analysis 2 of the Diagnostic Accuracy of Baseline Information (“No Test”) and of the Summary Scores for Home-Monitoring Tests to Identify the Reference Standard of Lesion Activity in Study Eyes Identified by Reading Center Grading, Using All Available Home-Monitoring Data **eTable 5.** Model Results for the Alternative Reference Standard: a Change From Inactive to Active Lesion Status **eTable 6.** MONARCH Diagnostic Test Accuracy Checklist for Adherence to STARD Reporting Guidelines

Supplement 2.
Data Sharing Statement.
