# Supplementary material for: Home-Monitoring Vision Tests to Detect Active Neovascular Age-Related Macular Degeneration
Source: JAMA Ophthalmol. 2024 Apr 25;142(6):512–20. doi: 10.1001/jamaophthalmol.2024.0918 (PMC11046404; doi:10.1001/jamaophthalmol.2024.0918)
Supplement: Supplement 1. — eFigure 1. Equipment Pack Given to Participants Included an iOS-Based Mobile Device, Eye Patch, Stylus, Screen Cloth, Laminated Instruction Card, Wireless Internet Device, 2 Charging Leads, and a Double USB Charger Plug eFigure 2. Receiver Operating Characteristic Curves and AUROCs (95% CIs) for Summary Test Scores for All Index Tests; Sensitivity Analysis 2 (Reference Standard Derived From Independent Grading of OCTs) eTable 1. Reasons for Being Unwilling in Principle to Participate eTable 2. Primary Analysis of the Diagnostic Accuracy of Baseline Information (“No Test”) and of the Summary Scores for Home-Monitoring Tests to Identify the Reference Standard of Lesion Activity in Study Eyes eTable 3. Model Results for Sensitivity Analysis 1 of the Diagnostic Accuracy of Baseline Information (“No Test”) and of the Summary Scores for Home-Monitoring Tests to Identify the Reference Standard of Lesion Activity in Study Eyes Used Home-Monitoring Data for Only the 4 Weeks Preceding the Hospital Monitoring Visit eTable 4. Model Results for Sensitivity Analysis 2 of the Diagnostic Accuracy of Baseline Information (“No Test”) and of the Summary Scores for Home-Monitoring Tests to Identify the Reference Standard of Lesion Activity in Study Eyes Identified by Reading Center Grading, Using All Available Home-Monitoring Data eTable 5. Model Results for the Alternative Reference Standard: a Change From Inactive to Active Lesion Status eTable 6. MONARCH Diagnostic Test Accuracy Checklist for Adherence to STARD Reporting Guidelines [file jamaophthalmol-e240918-s001.pdf]

Supplementary Online Content

Hogg RE, Sivaprasad S, Wickens R, et al. Home-monitoring vision tests to detect active neovascular age-related macular degeneration. *JAMA Ophthalmol*. Published online April 25, 2024. doi:10.1001/jamaophthalmol.2024.0918

eFigure 1. Equipment Pack Given to Participants Included an iOS-Based Mobile Device, Eye Patch, Stylus, Screen Cloth, Laminated Instruction Card, Wireless Internet Device, 2 Charging Leads, and a Double USB Charger Plug

eFigure 2. Receiver Operating Characteristic Curves and AUROCs (95% CIs) for Summary Test Scores for All Index Tests; Sensitivity Analysis 2 (Reference Standard Derived From Independent Grading of OCTs)

eTable 1. Reasons for Being Unwilling in Principle to Participate

eTable 2. Primary Analysis of the Diagnostic Accuracy of Baseline Information (“No Test”) and of the Summary Scores for Home-Monitoring Tests to Identify the Reference Standard of Lesion Activity in Study Eyes

eTable 3. Model Results for Sensitivity Analysis 1 of the Diagnostic Accuracy of Baseline Information (“No Test”) and of the Summary Scores for Home-Monitoring Tests to Identify the Reference Standard of Lesion Activity in Study Eyes Used Home-Monitoring Data for Only the 4 Weeks Preceding the Hospital Monitoring Visit

eTable 4. Model Results for Sensitivity Analysis 2 of the Diagnostic Accuracy of Baseline Information (“No Test”) and of the Summary Scores for Home-Monitoring Tests to Identify the Reference Standard of Lesion Activity in Study Eyes Identified by Reading Center Grading, Using All Available Home-Monitoring Data

eTable 5. Model Results for the Alternative Reference Standard: a Change From Inactive to Active Lesion Status

eTable 6. MONARCH Diagnostic Test Accuracy Checklist for Adherence to STARD Reporting Guidelines

This supplementary material has been provided by the authors to give readers additional information about their work.

© 2024 Hogg RE et al. *JAMA Ophthalmology*.

eFigure 1- Equipment pack given to participants included an iOS-Based Mobile Device (iPod Touch [Apple]), Eye patch, stylus, screen cloth, laminated instruction card, wireless internet device, 2 charging leads, and a double USB charger plug

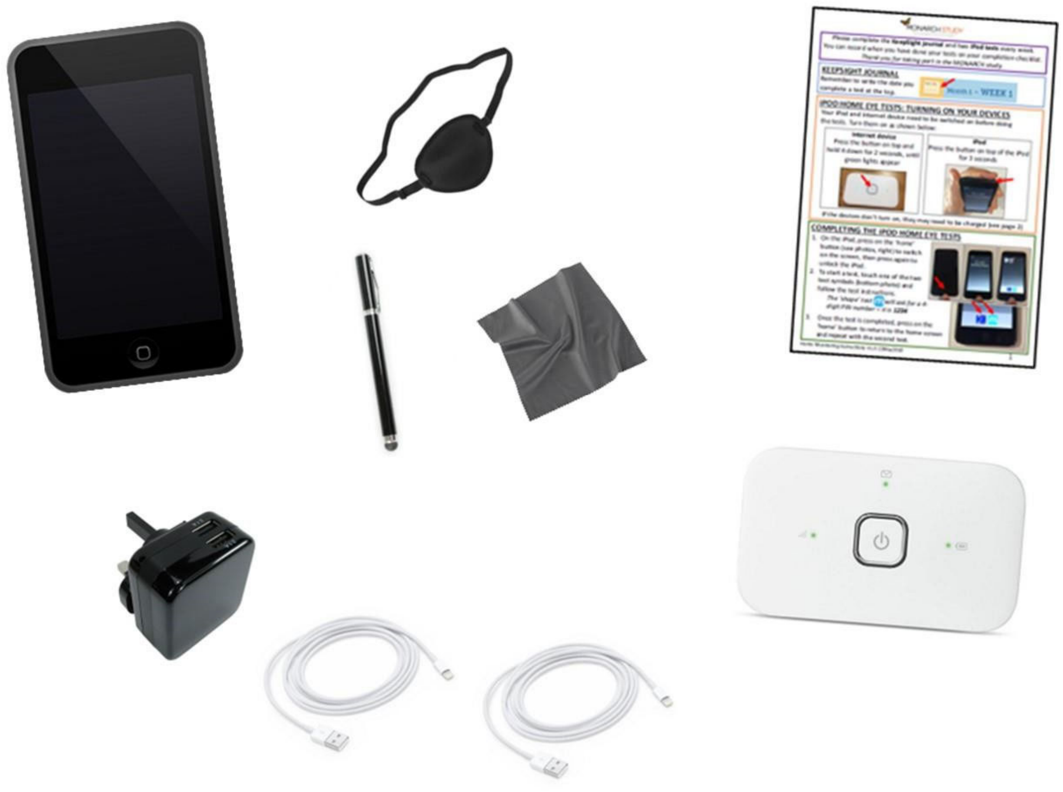

© 2024 Hogg RE et al. *JAMA Ophthalmology*.

eFigure 2 Receiver operating characteristic curves and AUROCs (95% CIs) for summary test scores for all index tests; sensitivity analysis 2 (reference standard derived from independent grading of OCTs).

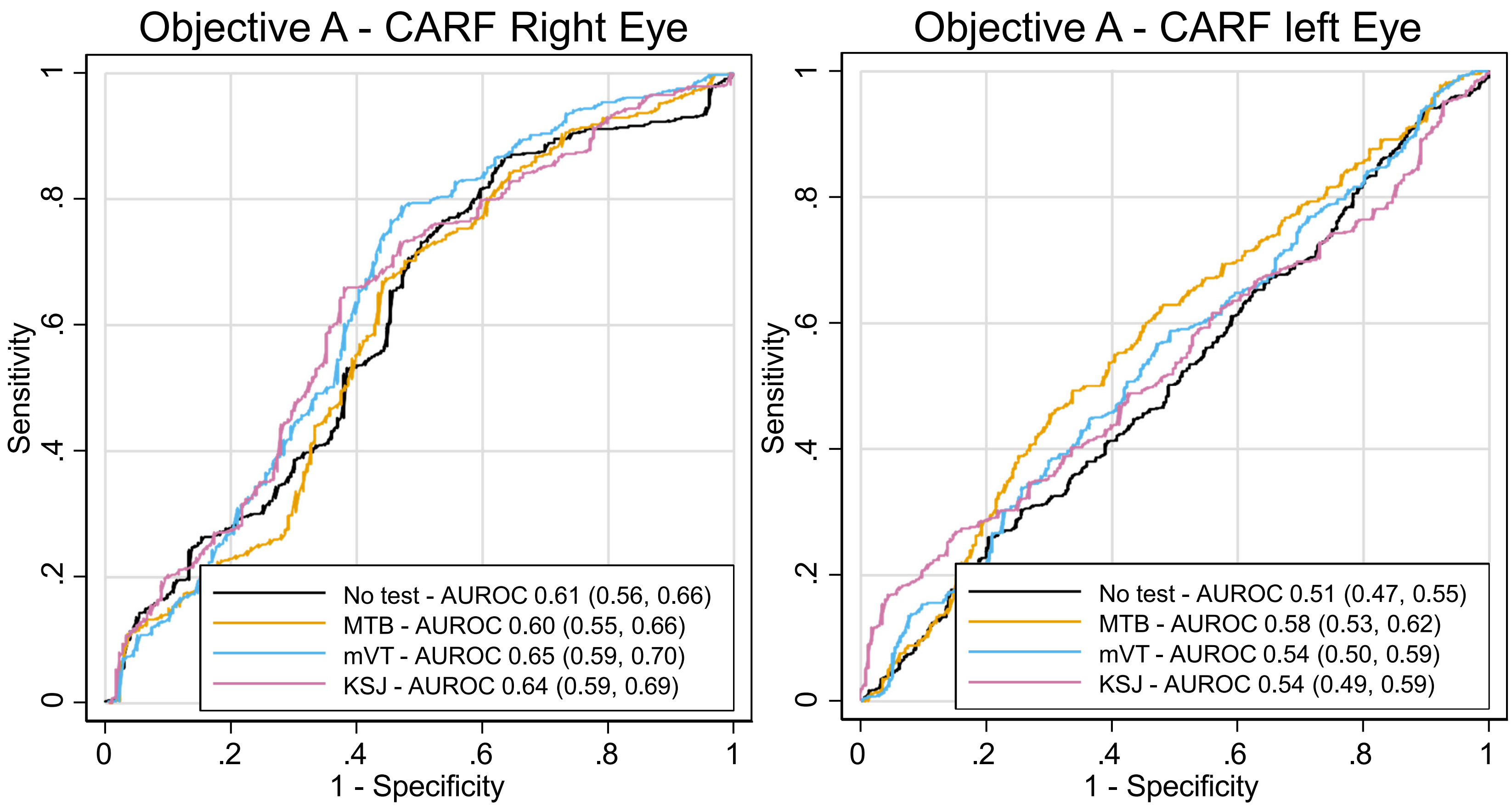

© 2024 Hogg RE et al. *JAMA Ophthalmology*.

eTable 1: Reasons for being unwilling in principle to participate.

**Reason for Not Willing**	**Overall n (%)**
Ineligible	**10 (2%)**
No reason given	**76 (15%)**
Not interested	**83 (17%)**
Put off by technology	**95 (19%)**
No benefit in taking part	**9 (2%)**
Personal reasons	**86 (17%)**
Not enough time to consider	**1 (0%)**
Insurance invalidated	**-- (--%)**
Unable to agree to consent questions	**1 (0%)**
Too much of a commitment	**54 (11%)**
Covid19 recruitment pause	**17 (3%)**
Medical Reason	**28 (5%)**
Too far to travel	**8 (2%)**
Feels too old	**8(2%)**
Not Interested	**9(2%)**
Other[1]	**17 (3%)**
**Overall (within site)**	**502 (100%)**

Other reasons include:
1 Poor memory, 1 Poor mobility, 1 Does not want internet, 4 Does not feel capable, 2 Lives alone, 1 Initially agreed, but changed mind, 2 Too complicated; 1 Not eligible, 1 Discharged from follow-up, 1 Did not attend training, 1 Poor mobile signal at home, 1 Patient died.

© 2024 Hogg RE et al. *JAMA Ophthalmology.*

eTable 2: Primary analysis of the diagnostic accuracy of baseline information ("no test") and of the summary scores for home-monitoring tests to identify the reference standard of lesion activity in study eyes. Each model was fitted, where possible, with a random intercept and random slope on calendar quarter since baseline visit at the participant level, and a random intercept at the eye level.

(a) Numbers of visits, eyes and participants contributing to the models and odds ratios for predictors in the models.

**Numbers in models**	**No Test**		**MTB**		**mVT**		**KSJ**	
Visits	1413		1213		1249		1238	
Participants	252		233		237		224	
Eyes	302		279		284		270	
**Predictor in model**	**OR (95% CI)**	***p***	**OR (95% CI)**	***p***	**OR (95% CI)**	***p***	**OR (95% CI)**	***p***
Mean MTB Score	-	-	1.02 (1.00, 1.04)	*0.083*	-	-	-	-
Mean mVT Score[1]	-	-	-	-	0.99 (0.98, 1.01)	*0.432*	-	-
Median KSJ VA Score	-	-	-	-	-	-	1.33 (0.97, 1.84)	*0.080*
% KSJ VA worse[2]	-	-	-	-	-	-	3.48 (1.09, 11.1)	*0.036*
% KSJ Amsler Grid worse[2]	-	-	-	-	-	-	3.39 (0.70, 16.5)	*0.130*
% KSJ Household Object worse[2]	-	-	-	-	-	-	1.14 (0.20, 6.54)	*0.880*
Sex								
Male	1		1		1		1	
Female	0.72 (0.29, 1.78)	*0.481*	0.73 (0.29, 1.81)	*0.496*	0.82 (0.33, 2.02)	*0.670*	0.51 (0.20, 1.29)	*0.153*
Time since first treatment at baseline strata								
6-17 months	1		1		1		1	
18-29 months	0.67 (0.25, 1.81)	*0.429*	0.96 (0.35, 2.64)	*0.943*	0.87 (0.32, 2.36)	*0.781*	0.60 (0.22, 1.68)	*0.331*
30-41 months	0.45 (0.15, 1.39)	*0.165*	0.50 (0.16, 1.55)	*0.228*	0.47 (0.15, 1.47)	*0.194*	0.54 (0.17, 1.70)	*0.290*
Age (per year)	0.99 (0.93, 1.06)	*0.744*	1.00 (0.93, 1.07)	*0.946*	0.98 (0.91, 1.05)	*0.537*	1.00 (0.94, 1.07)	*0.906*
Baseline VA Strata								
Better than or equal to 6/18	1		1		1		1	
Worse than 6/18 & better than 6/24	0.72 (0.15, 3.52)	*0.684*	1.86 (0.34, 10.12)	*0.472*	1.14 (0.22, 6.05)	*0.875*	1.00 (0.2, 5.03)	*0.997*
Worse than or equal to 6/24	0.64 (0.10, 4.10)	*0.638*	0.77 (0.11, 5.19)	*0.787*	0.91 (0.14, 5.93)	*0.918*	0.96 (0.14, 6.59)	*0.970*

© 2024 Hogg RE et al. *JAMA Ophthalmology.*

Days since baseline[3]	0.52 (0.36, 0.74)	*<0.001*	0.49 (0.33, 0.72)	*<0.001*	0.54 (0.37, 0.78)	*0.001*	0.52 (0.36, 0.75)	*<0.001*

1 Transformed by multiplying by 100

2 Proportion of scores across the respective interval that reported vision as being 'worse'

3 Natural log transformed

(b) Estimated AUROCs, Youden's indices and sensitivity, specificity, positive (PPV) and negative predictive value (NPV) at thresholds defined by Youden's index.

**Test**	**No test**	**MTB**	**mVT**	**KSJ**
AUROC (95% CI)	0.554 (0.525, 0.584)	0.586 (0.554, 0.619)	0.572 (0.540, 0.604)	0.574 (0.542, 0.606)
Youden's Index	0.09	0.14	0.12	0.13
Sensitivity	0.36	0.47	0.41	0.36
Specificity	0.73	0.67	0.72	0.77
PPV	0.66	0.68	0.68	0.70
NPV	0.44	0.46	0.45	0.45

AUROC – area under the ROC curve; PPV – positive predictive value; NPV – negative predictive value

(c) Average test scores above and below thresholds defined by Youden's index.

	**Below threshold**		**Above threshold**	
	n	**Average[1] (95%, CI)**	***n***	**Average[1] (95%, CI)**
**MTB**	711	81.31 (79.88, 82.75)	502	88.07 (87.03, 89.1)
**mVT**	804	-0.38 (-0.39, -0.36)	445	-0.47 (-0.49, -0.45)
**KSJ**				
VA (median, IQR)[2]	860	5 (5, 6)	378	5.25 (5, 6)
VA worse	860	0.08 (0.07, 0.09)	378	0.24 (0.2, 0.27)
Amsler Grid worse	860	0.06 (0.05, 0.06)	378	0.24 (0.21, 0.28)
Household object worse	860	0.04 (0.03, 0.05)	378	0.16 (0.13, 0.19)

1 Average scores below/above the threshold are means unless otherwise specified. KSJ mean summary scores are expressed the proportion who reported worse for the given test.

2 VA is on a self-reported scale where 1 = worst and 6 = best

© 2024 Hogg RE et al. *JAMA Ophthalmology.*

eTable 3: Model Results for sensitivity analysis 1 of the diagnostic accuracy of baseline information (“no test”) and of the summary scores for home-monitoring tests to identify the reference standard of lesion activity in study eyes used home-monitoring data for only the 4 weeks preceding the hospital monitoring visit. Each model was fitted, where possible, with a random intercept and random slope on calendar quarter since baseline visit at the participant level, and a random intercept at the eye level.

(a) Numbers of visits, eyes and participants contributing to the models and odds ratios for predictors in the models.

**Numbers in models**	**No Test**		**MTB**		**mVT**		**KSJ**	
Visits	1413		1213		1134		1173	
Participants	252		233		210		218	
Eyes	302		279		253		260	
**Predictor in model**	**OR (95% CI)**	***p***	**OR (95% CI)**	***p***	**OR (95% CI)**	***p***	**OR (95% CI)**	***p***
Mean MTB Score	-	-	1 (0.98, 1.03)	0.679	-	-	-	-
Mean mVT Score[1]	-	-	-	-	0.99 (0.98, 1.01)	0.379	-	-
Median KSJ VA Score	-	-	-	-	-	-	1.30 (0.94, 1.79)	0.112
% KSJ VA worse[2]	-	-	-	-	-	-	2.57 (0.99, 6.71)	0.053
% KSJ Amsler Grid worse[2]	-	-	-	-	-	-	2.10 (0.59, 7.47)	0.252
% KSJ Household Object worse[2]	-	-	-	-	-	-	2.38 (0.54, 10.56)	0.255
Sex								
Male	1		1		1		1	
Female	0.72 (0.29, 1.78)	*0.481*	0.8 (0.31, 2.07)	0.642	0.71 (0.29, 1.77)	0.466	0.57 (0.22, 1.47)	0.242
Time since first treatment at baseline strata								
6-17 months	1		1		1		1	
18-29 months	0.67 (0.25, 1.81)	*0.429*	0.96 (0.33, 2.77)	0.938	0.98 (0.35, 2.69)	0.961	0.54 (0.19, 1.54)	0.251
30-41 months	0.45 (0.15, 1.39)	*0.165*	0.44 (0.13, 1.48)	0.185	0.47 (0.15, 1.50)	0.201	0.57 (0.17, 1.88)	0.352
Age (per year)	0.99 (0.93, 1.06)	*0.744*	0.99 (0.92, 1.06)	0.776	0.98 (0.91, 1.05)	0.566	1.01 (0.94, 1.09)	0.712
Baseline VA Strata								
Better than or equal to 6/18	1		1		1		1	
Worse than 6/18 & better than 6/24	0.72 (0.15, 3.52)	*0.684*	1.10 (0.20, 6.06)	0.912	1.25 (0.24, 6.63)	0.791	0.71 (0.14, 3.63)	0.683
Worse than or equal to 6/24	0.64 (0.10, 4.10)	*0.638*	0.54 (0.08, 3.68)	0.531	0.82 (0.13, 5.31)	0.838	0.81 (0.12, 5.51)	0.827
Days since baseline[3]	0.52 (0.36, 0.74)	*<0.001*	0.48 (0.32, 0.73)	*<0.001*	0.54 (0.37, 0.79)	0.001	0.51 (0.34, 0.74)	0.001

© 2024 Hogg RE et al. *JAMA Ophthalmology.*

1. *Transformed by multiplying by 100*
2. *Proportion of scores across the respective interval that reported vision as being 'worse'*
3. *Natural log-transformed*

(b) Estimated AUROCs for sensitivity analysis 1: Youden's indices and sensitivity, specificity, positive (PPV) and negative predictive value (NPV) at thresholds defined by Youden's index.

Test	No test	MTB	mVT	KSJ
AUROC (95% CI)	0.554 (0.524, 0.584)	0.586 (0.554, 0.619)	0.576 (0.542, 0.609)	0.575 (0.542, 0.608)
Youden's Index	0.09	0.14	0.14	0.12
Sensitivity	0.36	0.47	0.46	0.47
Specificity	0.73	0.67	0.67	0.65
PPV	0.66	0.68	0.68	0.66
NPV	0.44	0.46	0.46	0.46

AUROC – area under the ROC curve; PPV – positive predictive value; NPV – negative predictive value

(c) Sensitivity analysis 1: Average test scores above and below thresholds defined by Youden's index.

	Below threshold		Above threshold	
	n	Average[1] (95%, CI)	*n*	Average[1] (95%, CI)
**MTB**	671	83.65 (82.30, 85.01)	463	85.4 (83.93, 86.88)
**mVT**	680	-0.36 (-0.38, -0.35)	493	-0.47 (-0.49, -0.45)
**KSJ**				
VA (median, IQR)[2]	985	5 (5, 6)	117	5.5 (5, 6)
VA worse	985	0.09 (0.08, 0.10)	117	0.31 (0.26, 0.36)
Amsler Grid worse	985	0.07 (0.06, 0.08)	117	0.35 (0.29, 0.40)
Household object worse	985	0.04 (0.03, 0.05)	117	0.25 (0.20, 0.30)

1 Average scores below/above the threshold are means unless otherwise specified. KSJ mean summary scores are expressed the proportion who reported worse for the given test.

2 VA is on a self-reported scale where 1 = worst and 6 = best

© 2024 Hogg RE et al. *JAMA Ophthalmology.*

eTable 4 Model Results for sensitivity analysis 2 of the diagnostic accuracy of baseline information ("no test") and of the summary scores for home-monitoring tests to identify the reference standard of lesion activity in study eyes identified by reading center grading, using all available home-monitoring data; models for right and left eyes fitted separately. Each model was fitted, where possible, with a random intercept and random slope on calendar quarter since baseline visit at the participant level.

(a) Model results for the primary outcome of lesion activity in **right** study eyes for all home monitoring test models and for a no-test model.

**Numbers in models**	**No Test**		**MTB**		**mVT**		**KSJ**	
Visits	643		561		583		552	
Participants	147		135		140		132	
**Predictor in model**	**OR (95% CI)**	***p***	**OR (95% CI)**	***p***	**OR (95% CI)**	***p***	**OR (95% CI)**	***p***
Mean MTB Score	-	-	1.05 (1, 1.1)	0.038*	-	-	-	-
Mean mVT Score[1]	-	-	-	-	0.98 (0.95, 1.00)	0.097	-	-
Median KSJ VA Score	-	-	-	-	-	-	1.49 (0.78, 2.83)	0.224
% KSJ VA worse[2]	-	-	-	-	-	-	0.62 (0.09, 4.37)	0.634
% KSJ Amsler Grid worse[2]	-	-	-	-	-	-	0.41 (0.03, 5.79)	0.507
% KSJ Household Object worse[2]	-	-	-	-	-	-	3.41 (0.20, 59.2)	0.399
Sex								
Male	1		1		1		1	
Female	0.58 (0.13, 2.65)	0.479	0.44 (0.09, 2.18)	0.312	0.56 (0.13, 2.42)	0.436	0.66 (0.13, 3.46)	0.622
Time since first treatment at baseline strata								
6-17 months	1		1		1		1	
18-29 months	2.91 (0.52, 16.1)	0.222	2.01 (0.34, 11.90)	0.441	2.95 (0.56, 15.6)	0.203	3.76 (0.57, 24.5)	0.167
30-41 months	1.41 (0.22, 9.22)	0.719	2.40 (0.33, 17.49)	0.386	2.08 (0.33, 13.0)	0.434	1.89 (0.25, 14.3)	0.538
Age (per year)	0.86 (0.76, 0.97)	0.015*	0.93 (0.82, 1.04)	0.206	0.89 (0.79, 1.00)	0.053	0.89 (0.79, 1.01)	0.070
Baseline VA Strata								
Better than or equal to 6/18	1		1		1		1	
Worse than 6/18 & better than 6/24	12.40 (0.35, 436)	0.166	25.9 (0.63, 1059)	0.086	16.3 (0.55, 484)	0.107	88.2 (1.28, 6077)	0.038
Worse than or equal to 6/24	0.81 (0.05, 12.5)	0.880	0.9 (0.05, 15.00)	0.940	1.66 (0.10, 27.6)	0.722	2.37 (0.09, 61.7)	0.603
Days since baseline[3]	1.10 (0.60, 2.02)	0.761	1.01 (0.54, 1.89)	0.970	1.22 (0.67, 2.23)	0.516	1.11 (0.55, 2.24)	0.769

1 Transformed by multiplying by 100

© 2024 Hogg RE et al. *JAMA Ophthalmology.*

2 Proportion of scores across the respective interval that reported vision as being 'worse'
3 Natural log transformed

Model results for the primary outcome of lesion activity in **left** study eyes for all home monitoring test models and for a no-test model.

**Numbers in models**	**No Test**		**MTB**		**mVT**		**KSJ**	
Visits	716		607		620		632	
Participants	148		136		137		130	
**Predictor in model**	**OR (95% CI)**	***p***	**OR (95% CI)**	***p***	**OR (95% CI)**	***p***	**OR (95% CI)**	***p***
Mean MTB Score	-	-	0.98 (0.94, 1.02)	0.366	-	-	-	-
Mean mVT Score[1]	-	-	-	-	1.04 (1.01, 1.08)	0.015	-	-
Median KSJ VA Score	-	-	-	-	-	-	0.87 (0.44, 1.73)	0.689
% KSJ VA worse[2]	-	-	-	-	-	-	1.56 (0.14, 17.3)	0.719
% KSJ Amsler Grid worse[2]	-	-	-	-	-	-	2.52 (0.08, 77.2)	0.596
% KSJ Household Object worse[2]	-	-	-	-	-	-	3.93 (0.06, 240)	0.515
Sex								
Male	1		1		1		1	
Female	1.44 (0.26, 7.87)	0.677	1.19 (0.22, 6.28)	0.841	1.14 (0.21, 6.05)	0.882	1.83 (0.31, 10.9)	0.507
Time since first treatment at baseline strata								
6-17 months	1		1		1		1	
18-29 months	0.52 (0.08, 3.34)	0.490	1.26 (0.20, 7.81)	0.803	0.50 (0.08, 3.18)	0.464	0.48 (0.07, 3.28)	0.452
30-41 months	0.44 (0.05, 4.20)	0.473	0.60 (0.07, 5.17)	0.645	0.28 (0.03, 2.53)	0.258	0.51 (0.05, 5.52)	0.582
Age	1.08 (0.95, 1.23)	0.244	1.05 (0.92, 1.20)	0.441	1.04 (0.91, 1.18)	0.566	1.04 (0.91, 1.18)	0.598
Baseline VA Strata								
Better than or equal to 6/18	1		1		1		1	
Worse than 6/18 & better than 6/24	3.52 (0.17, 74.2)	0.418	8.90 (0.19, 415)	0.265	0.8 (0.03, 24.82)	0.896	1.45 (0.06, 35.01)	0.820
Days since baseline[3]	0.68 (0.35, 1.31)	0.249	0.66 (0.36, 1.20)	0.174	0.78 (0.42, 1.43)	0.418	0.81 (0.38, 1.72)	0.588

1 Transformed by multiplying by 100
2 Proportion of scores across the respective interval that reported vision as being 'worse'

© 2024 Hogg RE et al. *JAMA Ophthalmology.*

3 Natural log transformed

(b) Model performance for predicting management decisions of lesion activity at thresholds defined by Youden's index for summary test scores for all index tests; sensitivity analysis 2.

Right eyes

Test	No test	MTB	mVT	KSJ
AUROC (95% CI)	0.611 (0.563, 0.659)	0.602 (0.548, 0.656)	0.646 (0.593, 0.698)	0.636 (0.585, 0.686)
Youden's Index	0.23	0.23	0.32	0.28
Sensitivity	0.86	0.67	0.79	0.66
Specificity	0.37	0.56	0.53	0.62
PPV	0.75	0.78	0.79	0.78
NPV	0.55	0.42	0.52	0.47

AUROC – area under the ROC curve; PPV – positive predictive value; NPV – negative predictive value

Left eyes

Test	No test	MTB	mVT	KSJ
AUROC (95% CI)	0.5111 (0.4676, 0.5547)	0.5767 (0.5294, 0.624)	0.5427 (0.4961, 0.5893)	0.5359 (0.4908, 0.5811)
Youden's Index	0.06	0.15	0.1	0.12
Sensitivity	0.26	0.49	0.57	0.17
Specificity	0.8	0.66	0.53	0.96
PPV	0.68	0.72	0.67	0.88
NPV	0.39	0.42	0.42	0.38

AUROC – area under the ROC curve; PPV – positive predictive value; NPV – negative predictive value

(c) Average test scores above and below thresholds defined by Youden's index for the models predicting management decisions of lesion activity for all index tests; sensitivity analysis 2.

© 2024 Hogg RE et al. *JAMA Ophthalmology.*

Right eyes	Below threshold		Above threshold	
	n	Average[1] (95%, CI)	*n*	Average[1] (95%, CI)
**MTB**	224	74 (71.49, 76.51)	337	91.09 (89.94, 92.24)
**mVT**	179	-0.26 (-0.29, -0.22)	404	-0.46 (-0.48, -0.44)
**KSJ**				
VA (median, IQR)[2]	245	5 (4, 6)	316	6 (5, 6)
VA worse	245	0.18 (0.15, 0.22)	316	0.1 (0.08, 0.12)
Amsler grid worse	245	0.19 (0.15, 0.23)	316	0.08 (0.06, 0.10)
Household object worse	245	0.13 (0.09, 0.16)	316	0.07 (0.05, 0.09)

1 Average scores below/above the threshold are means unless otherwise specified. KSJ mean summary scores are expressed the proportion who reported worse for the given test.

2 VA is on a self-reported scale where 1 = worst and 6 = best

Left eyes	Below threshold		Above threshold	
	n	Average[1] (95%, CI)	*n*	Average[1] (95%, CI)
**MTB**	348	91.99 (91.16, 92.83)	270	74.22 (71.87, 76.56)
**mVT**	296	-0.59 (-0.60, -0.57)	335	-0.28 (-0.30, -0.26)
**KSJ**				
VA (median, IQR)[2]	952	6 (5, 6)	85	5 (3, 5)
VA worse	952	0.08 (0.07, 0.10)	85	0.35 (0.27, 0.43)
Amsler grid worse	952	0.05 (0.04, 0.06)	85	0.45 (0.37, 0.52)
Household object worse	952	0.03 (0.02, 0.03)	85	0.31 (0.24, 0.39)

1 Average scores below/above the threshold are means unless otherwise specified. KSJ mean summary scores are expressed the proportion who reported worse for the given test.

2 VA is on a self-reported scale where 1 = worst and 6 = best

© 2024 Hogg RE et al. *JAMA Ophthalmology.*

eTable 5 Model results for the alternative reference standard: a change from inactive to active lesion status. Each model was fitted, where possible, with a random intercept and random slope on calendar quarter since baseline visit at the participant level, and a random intercept at the eye level.

(a) Model results for all home monitoring test models and for a no-test model

	**No Test**		**MTB**		**KSJ**	
Visit pairs	1413		1213		1238	
Inactive to active transitions	131 (9.3%)		104 (8.6%)		120 (9.7%)	
Participants	252		233		224	
**Predictor in model**	**OR (95% CI)**	***p***	**OR (95% CI)**	***p***	**OR (95% CI)**	***p***
Mean MTB Score	-	-	1.00 (0.99, 1.02)	0.601	-	-
Median KSJ VA Score	-	-	-	-	1.11 (0.89, 1.38)	0.372
% KSJ VA worse[1]	-	-	-	-	1.21 (0.43, 3.40)	0.715
% KSJ Amsler Grid worse[1]	-	-	-	-	0.64 (0.15, 2.71)	0.543
% KSJ Household Object worse[1]	-	-	-	-	1.82 (0.37, 9.05)	0.464
Sex						
Male	1					
Female	1.05 (0.69, 1.61)	*0.815*	1.18 (0.75, 1.84)	*0.475*	1.00 (0.64, 1.54)	*0.989*
Time since first treatment at baseline strata						
6-17 months						
18-29 months	0.79 (0.49, 1.28)	*0.349*	0.76 (0.45, 1.26)	*0.284*	0.81 (0.50, 1.29)	*0.37*
30-41 months	0.95 (0.54, 1.68)	*0.860*	0.96 (0.53, 1.75)	*0.903*	0.91 (0.50, 1.64)	*0.747*
Age (per year)	1.02 (0.99, 1.05)	*0.190*	1.02 (0.99, 1.06)	*0.194*	1.03 (0.996, 1.06)	*0.091*
Baseline VA Strata						
Better than or equal to 6/18						
Worse than 6/18 & better than 6/24	0.56 (0.21, 1.52)	*0.255*	0.77 (0.28, 2.17)	*0.625*	0.66 (0.24, 1.85)	*0.428*
Worse than or equal to 6/24	0.93 (0.31, 2.82)	*0.904*	0.80 (0.23, 2.82)	*0.727*	1.06 (0.33, 3.42)	*0.928*
Days since baseline	1.001 (0.999, 1.002)	*0.236*	1.001 (1.000, 1.003)	*0.112*	1.001 (0.999, 1.002)	*0.452*

mVT test not shown because no model achieved convergence

1 Proportion of scores across the respective interval that reported vision as being 'worse'

© 2024 Hogg RE et al. *JAMA Ophthalmology.*

(b) Model performance for predicting management decisions for thresholds defined by Youden's index for summary test scores for index tests (except for the mVT, for which no model achieved convergence)

Test	No test	MTB	KSJ
AUROC (95% CI)	0.5686 (0.5165, 0.6208)	0.5807 (0.5244, 0.6371)	0.5806 (0.5253, 0.636)
Youden's Index	0.13	0.17	0.17
Sensitivity	0.63	0.65	0.4
Specificity	0.50	0.51	0.77
PPV	0.11	0.11	0.16
NPV	0.93	0.94	0.92

AUROC – area under the ROC curve; PPV – positive predictive value; NPV – negative predictive value

(c) Average test scores above and below thresholds defined by Youden's index for the models predicting management decisions tests (except for the mVT, for which no model achieved convergence)

	Below threshold		Above threshold	
	n	**Average[1] (95%, CI)**	***n***	**Average[1] (95%, CI)**
**MTB**	604	81.23 (79.69, 82.78)	609	86.96 (85.85, 88.07)
**KSJ**				
VA (median, IQR)	931	5 (5, 6)	307	6 (5, 6)
Comp	931	0.11 (0.10, 0.13)	307	0.16 (0.13, 0.19)
Object	931	0.06 (0.05, 0.07)	307	0.13 (0.10, 0.17)
Grid	931	0.10 (0.09, 0.12)	307	0.14 (0.11, 0.17)

1 Average scores below/above the threshold are means unless otherwise specified. KSJ mean summary scores are expressed the proportion who reported worse for the given test.

2 VA is on a self-reported scale where 1 = worst and 6 = best

© 2024 Hogg RE et al. *JAMA Ophthalmology.*

eTable 6- MONARCH Diagnostic Test Accuracy Chek-list for adherence to STARD reporting guidelines.

Section & Topic	No	Item	Reported on page #
**TITLE OR ABSTRACT**			
	**1**	Identification as a study of diagnostic accuracy using at least one measure of accuracy (such as sensitivity, specificity, predictive values, or AUC)	Title and abstract
**ABSTRACT**			
	**2**	Structured summary of study design, methods, results, and conclusions (for specific guidance, see STARD for Abstracts)	Abstract
**INTRODUCTION**			
	**3**	Scientific and clinical background, including the intended use and clinical role of the index test	6-7
	**4**	Study objectives and hypotheses	6
**METHODS**			
Study design	**5**	Whether data collection was planned before the index test and reference standard were performed (prospective study) or after (retrospective study)	7 (study design)
Participants	**6**	Eligibility criteria	7 (participants)
	**7**	On what basis potentially eligible participants were identified (such as symptoms, results from previous tests, inclusion in registry)	7 (participants)
	**8**	Where and when potentially eligible participants were identified (setting, location and dates)	7 (patient identification)
	**9**	Whether participants formed a consecutive, random or convenience series	8
Test methods	**10a**	Index test, in sufficient detail to allow replication	8-9
	**10b**	Reference standard, in sufficient detail to allow replication	9
	**11**	Rationale for choosing the reference standard (if alternatives exist)	Not applicable
	**12a**	Definition of and rationale for test positivity cut-offs or result categories of the index test, distinguishing pre-specified from exploratory	11 (AUROCs - cut-offs not available)
	**12b**	Definition of and rationale for test positivity cut-offs or result categories of the reference standard, distinguishing pre-specified from exploratory	9
	**13a**	Whether clinical information and reference standard results were available to the performers/readers of the index test	Reference standard identified after tests had been done
	**13b**	Whether clinical information and index test results were available to the assessors of the reference standard	9

© 2024 Hogg RE et al. *JAMA Ophthalmology.*

Analysis	**14**	Methods for estimating or comparing measures of diagnostic accuracy	Not applicable
	**15**	How indeterminate index test or reference standard results were handled	9
	**16**	How missing data on the index test and reference standard were handled	10
	**17**	Any analyses of variability in diagnostic accuracy, distinguishing pre-specified from exploratory	AUROCs presented. Figure 4
	**18**	Intended sample size and how it was determined	9-10
**RESULTS**			
Participants	**19**	Flow of participants, using a diagram	Figure 3
	**20**	Baseline demographic and clinical characteristics of participants	Table 1
	**21a**	Distribution of severity of disease in those with the target condition	Table 1
	**21b**	Distribution of alternative diagnoses in those without the target condition	Not applicable
	**22**	Time interval and any clinical interventions between index test and reference standard	Variable; addresses by sensitivity analysis 1
Test results	**23**	Cross tabulation of the index test results (or their distribution) by the results of the reference standard	Figure 4 (AUROCs) and Supplementary Table 2c
	**24**	Estimates of diagnostic accuracy and their precision (such as 95% confidence intervals)	Figure 4 (AUROCs) and Supplementary Table 2c
	**25**	Any adverse events from performing the index test or the reference standard	13
**DISCUSSION**			
	**26**	Study limitations, including sources of potential bias, statistical uncertainty, and generalisability	15
	**27**	Implications for practice, including the intended use and clinical role of the index test	16
**OTHER INFORMATION**			
	**28**	Registration number and name of registry	2
	**29**	Where the full study protocol can be accessed	NIHR Library webpage; ref 12
	**30**	Sources of funding and other support; role of funders	2; 7

© 2024 Hogg RE et al. *JAMA Ophthalmology.*

STARD 2015

AIM

STARD stands for "Standards for Reporting Diagnostic accuracy studies". This list of items was developed to contribute to the completeness and transparency of reporting of diagnostic accuracy studies. Authors can use the list to write informative study reports. Editors and peer-reviewers can use it to evaluate whether the information has been included in manuscripts submitted for publication.

EXPLANATION

A **diagnostic accuracy study** evaluates the ability of one or more medical tests to correctly classify study participants as having a **target condition.** This can be a disease, a disease stage, response or benefit from therapy, or an event or condition in the future. A medical test can be an imaging procedure, a laboratory test, elements from history and physical examination, a combination of these, or any other method for collecting information about the current health status of a patient.

The test whose accuracy is evaluated is called **index test.** A study can evaluate the accuracy of one or more index tests. Evaluating the ability of a medical test to correctly classify patients is typically done by comparing the distribution of the index test results with those of the **reference standard**. The reference standard is the best available method for establishing the presence or absence of the target condition. An accuracy study can rely on one or more reference standards.

If test results are categorized as either positive or negative, the cross tabulation of the index test results against those of the reference standard can be used to estimate the **sensitivity** of the index test (the proportion of participants *with* the target condition who have a positive index test), and its **specificity** (the proportion *without* the target condition who have a negative index test). From this cross tabulation (sometimes referred to as the contingency or "2x2" table), several other accuracy statistics can be estimated, such as the positive and negative **predictive values** of the test. Confidence intervals around estimates of accuracy can then be calculated to quantify the statistical **precision** of the measurements.

If the index test results can take more than two values, categorization of test results as positive or negative requires a **test positivity cut-off**. When multiple such cut-offs can be defined, authors can report a receiver operating characteristic (ROC) curve which graphically represents the combination of sensitivity and specificity for each possible test positivity cut-off. The **area under the ROC curve** informs in a single numerical value about the overall diagnostic accuracy of the index test.

The **intended use** of a medical test can be diagnosis, screening, staging, monitoring, surveillance, prediction or prognosis. The **clinical role** of a test explains its position relative to existing tests in the clinical pathway. A replacement test, for example, replaces an existing test. A triage test is used before an existing test; an add-on test is used after an existing test.

© 2024 Hogg RE et al. *JAMA Ophthalmology.*

Besides diagnostic accuracy, several other outcomes and statistics may be relevant in the evaluation of medical tests. Medical tests can also be used to classify patients for purposes other than diagnosis, such as staging or prognosis. The STARD list was not explicitly developed for these other outcomes, statistics, and study types, although most STARD items would still apply.

DEVELOPMENT

This STARD list was released in 2015. The 30 items were identified by an international expert group of methodologists, researchers, and editors. The guiding principle in the development of STARD was to select items that, when reported, would help readers to judge the potential for bias in the study, to appraise the applicability of the study findings and the validity of conclusions and recommendations. The list represents an update of the first version, which was published in 2003.

More information can be found on http://www.equator-network.org/reporting-guidelines/stard.

© 2024 Hogg RE et al. *JAMA Ophthalmology.*
