# Supplementary material for: Home-Monitoring Vision Tests to Detect Active Neovascular Age-Related Macular Degeneration
Source: JAMA Ophthalmol. 2024 Apr 25;142(6):512–20. doi: 10.1001/jamaophthalmol.2024.0918 (PMC11046404; doi:10.1001/jamaophthalmol.2024.0918)
Supplement: Supplement 2. — Data Sharing Statement. [file jamaophthalmol-e240918-s002.pdf]

Data Sharing Statement

Hogg. Home-Monitoring Vision Tests to Detect Active Neovascular Age-Related Macular Degeneration. *JAMA Ophthalmol*. Published April 25, 2024.
doi:10.1001/jamaophthalmol.2024.0918

Data
Data available: No

Additional Information
Explanation for why data not available: De-identified participant data can be made available upon request directed to the corresponding author. Proposals will be reviewed based on the proposed scientific objectives. An institutional review board approval and data user agreement will be required before the release of participant data.
